# Efficacy of urination in alleviating man’s urethral pain associated with flexible cystoscopy: a single-center randomized trial

**DOI:** 10.1186/s12894-019-0541-x

**Published:** 2020-01-20

**Authors:** Yingwei Xie, Wei Wang, Wei Yan, Dan Liu, Yuexin Liu

**Affiliations:** Department of Urology, Beijing Tongren Hospital, Capital Medical University, Beijing, 100730 China

**Keywords:** Flexible cystoscopy, Bladder, Pain

## Abstract

**Background:**

This study aimed to assess whether urethral pain can be alleviated by urination in male patients undergoing flexible cystoscopy.

**Methods:**

Ninety-six male outpatients undergoing flexible cystoscopy were randomly divided into two groups. Patients in the test group urinated during flexible cystoscopy, whilst patients in the control group received no instructions to do so. All patients received 10 mL of 2% lidocaine gel prior to assessment. Using 0 (no-pain) to 10 (unbearable severe pain) pain scores (VAS), we assessed patient discomfort prior to anesthesia gel perfusion (baseline), during gel perfusion, during cystoscope insertion through the urethra, and 15 min post-examination analysis. The entire protocol was completed by a single doctor in our Department of Urology.

**Results:**

The groups showed no statistical differences regarding age or examination time. During cystoscope insertion, the test group recorded significantly lower pain scores 2 (IQR 1–3) - compared to the control group 3 (IQR 2–3), (*P* = 0.001). No significant differences between other evaluation points were observed between groups.

**Conclusion:**

Urethral pain can be significantly alleviated by urination in male patients undergoing flexible cystoscopy through the urethra.

**Trial registration:**

**Registry name**: Clinical study of urination action to relieve urethral pain associated with flexible cystoscopy.

**Registration number**: ChiCTR-INR-17013294

**Date of Registration**: 2017-11-08

## Background

Cystoscopy is one of the most important tools for disease diagnosis in urology. Since its inception in 1984, flexible cystoscopy has been the procedure of choice for this purpose. Compared to rigid cystoscopes, flexible cystoscopy has the advantage of no blind areas, small damage, clear vision and a low requirement for body positioning. During analysis, the cystoscope must pass through the urethra to reach the bladder resulting in contact with the urethra mucosal membranes, leading to discomfort and pain. Patients receiving this procedure are thus often stressed, have increased heart rates and elevated blood pressure [1, 2].

Methods to alleviate pain in patients undergoing flexible cystoscopy have been widely investigated. Local anesthesia is a widely used analgesic method in flexible cystoscopy. Its analgesic theory is to inject anesthetic and penetrating drugs into the urethra to block the nerve located under the mucosa, thus playing the role of anesthesia. Local anesthetics are mostly gelatinous mixtures of anesthetics and lubricants, such as lidocaine gel, bupivacaine gel, tetracaine gel, etc. These anesthetic gels can not only play an anesthetic role in the urethra but also have lubrication. Lidocaine is currently the most widely used local anesthetic. However, recent studies have shown that lidocaine gel has no obvious advantage over urethra pain control compared with ordinary gel, and may cause additional pain and discomfort [3–5]. In addition to the use of anesthetics to relieve pain, more and more attention has been paid to the study of non-drug analgesic methods in recent years. There are many studies to alleviate pain through psychological or behavioral interventions. For example, watching the examination process through closed-circuit television [6, 7], listening to music [8, 9], etc.

We believe that defining the specific site of urethral pain caused by cystoscopy can help us control the pain more effectively. Chen [10] and Taghizadeh [11] have demonstrated that the most painful part of flexible cystoscopy is when the tip of the cystoscope passes through the external sphincter. Anatomically, the external urethral sphincter is located in the urethral membrane of the urethral stricture, and is also the most abundant area of afferent nerves. During urine storage, the external urethral sphincter is in a state of contraction and closes the membranous urethra. During urine passage, the external urethral sphincter relaxes [12].

We thus assumed that during the course of flexible cystoscopy, if the patient is urinating according to defined instructions, the external sphincter can relax, thereby reducing urethral pain during cystoscope insertion. Based on this hypothesis, we designed a prospective, randomized, controlled trial in which we assessed the levels of pain in patients instructed to urinate during flexible cystoscopy.

## Methods

### Ethical approval and trial registration

The trial was conducted between December 2017 and April 2018 in the Beijing Tongren Hospital in Beijing, China. The study was approved by the ethics committee of the Beijing Tongren Hospital, Capital Medical University (approval number: TRECKY2017–034) and registered at chictr.org.cn (registry number: ChiCTR-INR-17013294, date of registration 08 November 2017), prior to patient recruitment. The study was conducted according to common standard guidelines for clinical trials (Declaration of Helsinki, and the International Conference on Harmonization of Technical Requirements for Registration of Pharmaceuticals for Human Use and Good Clinical Practice (ICH-GCP) revised version, Somerset West, Republic of South Africa, 1996) [13]. All patients involved in this study gave their informed consent.

### Patients

Male patients were admitted to our clinic for flexible cystoscopy. All patients were over 18 years old. Exclusion criteria included any analgesic used within 24 h of the study period, known urethral stricture, previous history of urethral dilation, acute urinary tract infection, indwelling urethral catheter, and existing urethral pain (including chronic pelvic inflammatory disease and interstitial cystitis) Patients, as well as those who are unable to cope with pain assessment due to mental disorders.

### Randomization

Patients were screened by the chief physician of the outpatient clinic. Use a computer (Random Allocation Software, version 1.0.0) to build a random number table, then randomly divide patients into two groups. The randomisation was carried out by an independent researcher who was not involved in the subject-recruitment process. The sealed opaque envelopes were used to ensure concealed allocation. Intervention participants in the trial knew the patient’s grouping, and information collectors did not know the patient’s grouping.

### Design

We performed a prospective, randomized, controlled trial designed to compare pain scores between patients in experimental- and controls groups. Patients in the test group urinated during flexible cystoscopy. Patients in the control group received no instructions during the procedure. During examination, 10mls of 2% lidocaine gel was injected into the urethra and the penis was clamped for 10 min. A flexible cystoscope was passed through the urethra into the bladder for examination. All cystoscopies were performed by an experienced urologist. Flexible cystoscopy instruments included digital camera (Olympus OTV-S7), color video monitor (Olympus OEV-191H) and 16F flexible cystoscope (Olympus CYF-5A).

### Intervention

The patient was taken supine position during cystoscopy. Routine disinfection. During examination, 10mls of 2% lidocaine gel was injected into the urethra and the penis was clamped for 10 min. The procedure of the control group was as follows: the examiner lifted the penis with his left hand, inserted the front end of the flexible cystoscope into the urethra with the help of his left thumb and index finger, and held the mirror with his right hand. The thumb controlled the adjusting lever. With the help of water flow, the examiner observed the urethral side entry mirror under video surveillance. Stay 1–2 s when seeing the external urethral sphincter and entered the bladder under direct vision. In the experimental group, the examiner lifts the penis with his left hand, inserts the front end of the flexible cystoscope into the urethra with the help of his left thumb and index finger, holds the mirror with his right hand, and controls the control lever with his thumb. With the help of water flow, the examiner observed the urethral side entry mirror under video surveillance. Stay for 1–2 s when seeing the external urethral sphincter and ask the patient to urinate. Then entered the bladder under direct vision.

### Assessment

Patient histories were collected prior to operation. During cystoscopy, an experienced urologist nurse recorded the pain score according to the patient’s assessment before aesthesia gel perfusion (baseline), anaesthetized gel perfusion, cystoscopy insertion into the urethra, and 15 min post-examination analysis. Patients were asked to cross a line in a 10 cm region with 0 at one end of the transverse line indicating no pain; and 10 at the other end indicating a sharp intense pain.

### Collection of co-variate data

Before the experiment, researchers collected general information about patients, including age, height, weight, and underlying diseases.

### Sample size calculation

We used the result of Taghizadeh as the pain score of our control group, 2.82 ± 1.2VAS [11]. A minimal clinically important difference according to Todd was1.3 VAS [14]. Given the effect size of Cohen’s d = 0.615, and a two-sided 5% level t-test with a statistical power of 1-β = 80%, 43 patients would be needed to detect this group difference. We planned to include 96 patients in this trial (*n* = 48 per group); recognizing a potential loss of analytical power due to patient withdrawal from 10%.

### Data analyses

SPSS20.0 software was used for all data analysis. Data are expressed as mean ± standard deviation (SD), median and interquartile range (IQR) for variables that do not follow a normal distribution, or frequencies for categorical data. Normally distributed continuous variables were compared between the two groups using a two-sided t-test, with chi-square tests for categorical variables and with non-parametric Wilcoxon test for not normally distributed continuous variables. A *p*-value less than 0.05 was considered to be statistically significant.

## Results

The trial included 96 patients (48 cases in each group). As shown in Table 1, the average age of the test group was 53.35 ± 15.87 years, whilst the control group had an average age of 54.10 ± 15.48 years, which did not significantly differ (*P* = 0.815). Study groups did not differ in demographics, co-morbidities. The mean examination times were 3.42 ± 0.64 mins for the test group and 3.54 ± 0.71 mins for the control group, which did not significantly differ (*P* = 0.371).

**Table 1:** Patient data obtained during the study protocol

	Test group (*n* = 48)	Test group (*n* = 48)	*P* value
Years^a^	53.35 ± 15.87	54.10 ± 15.48	0.815
Mean Body mass index^a^	26 ± 3	26 ± 3	0.142
Hypertension n (%)	15 (31)	13 (27)	0.156
Diabetes Mellitus n (%)	7 (15)	11 (23)	0.122
Examination times (min)^a^	3.42 ± 0.64	3.54 ± 0.71	0.371

^a^Data presented as mean ± SD for normally distributed data or as percentage for categorical data

**Fig. 1:**
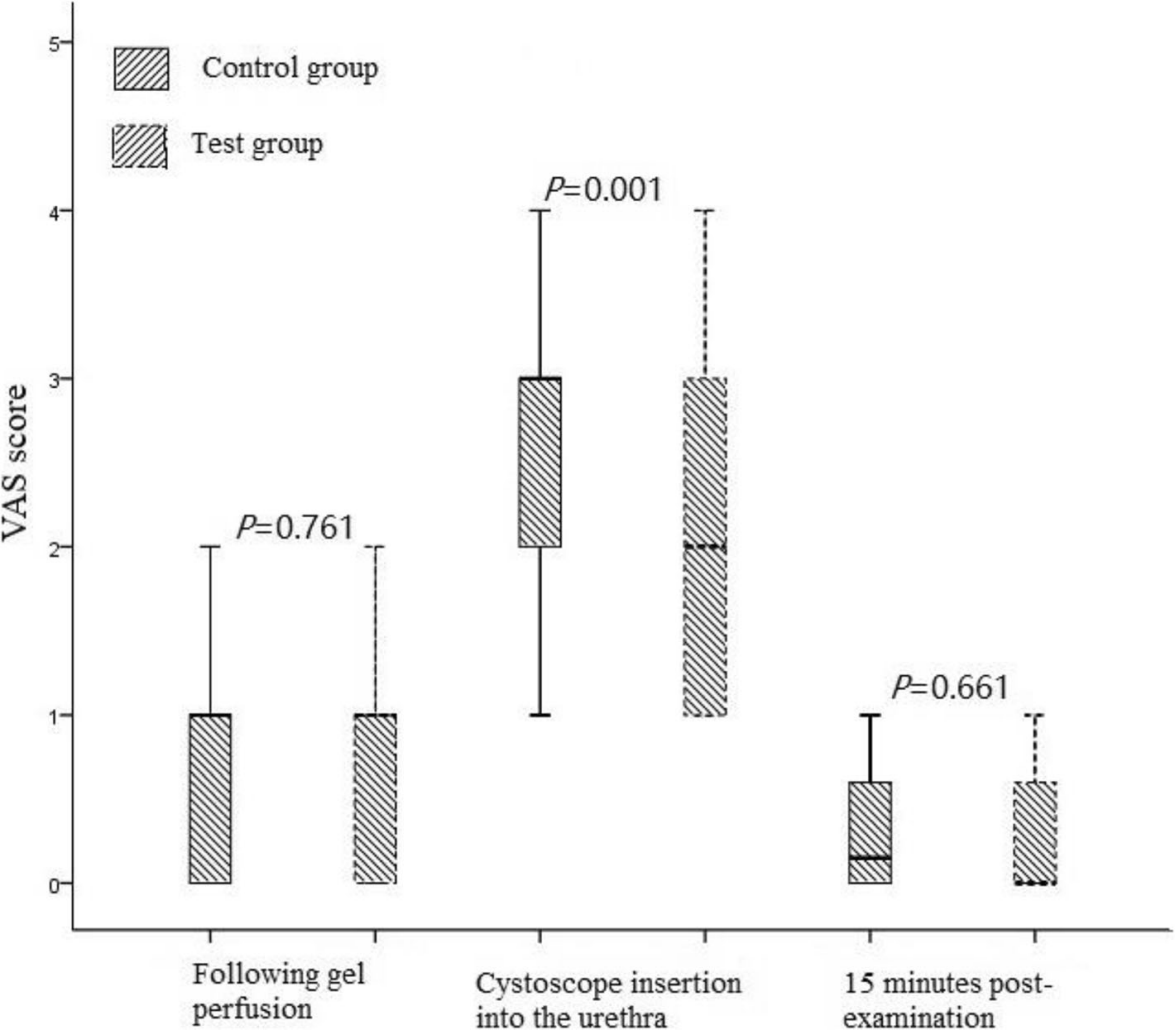
Comparison of the pain scores between the 2 groups at different checkpoints during cystoscopy

Regarding the VAS scores, no obvious urethral pain discomfort prior to anesthesia gel perfusion (baseline) was observed in either group (all scores = 0). When the two groups were compared after gel perfusion and 15 min following examination, no significant differences were observed (*P* = 0.761 & *P* = 0.661, respectively). Upon comparison of the scoring when the cystoscope was inserted, the score of the test group was 2 (IQR 1–3) compared to 3 (IQR 2–3) for the control group, which was significantly lower (*P* = 0.001) (Fig. 1).

## Discussion

Cystoscopy is routinely performed during urology assessments, particularly following bladder cancer surgery. It is therefore important to minimize the pain caused by this examination procedure. The invention of a flexible cystoscope greatly reduced the discomfort experienced during a patient’s examination and improved patient tolerance. However, pain was still unavoidable. Topical local anesthesia is currently widely employed but no consensus on the dosage, temperature, onset time and efficacy of its use have been assessed [4, 15]. In addition to the use of lubricants, more attention has been paid to non-pharmacological analgesic methods in recent years, including psychological and behavioral interventions. Patel & Zhang demonstrated that watching the examination process through closed-circuit television relieves urethral pain [6, 7], but these findings were refuted in later studies [16, 17]. Gunendran and coworkers found that squeezing brine bags significantly reduced pain and recommended the routine application of this procedure in males [18]. Zhang & Raheem also demonstrated that when patients listened to music, reduced pain and anxiety were observed [8, 9].

Due to the different urethral anatomy characteristics of male and female patients, the location and degree of pain caused by cystoscopy differs. When cystoscopy is performed, obvious resistance in the male urethra is sensed, particularly when the catheter passes through the urethral membrane. Taghizadeh and colleagues performed assessments in male patients in which they allowed patients to squeeze a balloon filled with water during contact with various areas of the urethra, evaluating the patient pain by the levels of water ejected. The results showed that cystoscopy caused the most significant pain when contacting the urethral membrane, where an average pain score of 2.82 was recorded. The second highest pain score occurred when the anaesthetized gel was squeezed into the urethra, displaying an average pain score of 0.84 [11]. This study provided direction for the future assessment of new analgesic methods for cystoscopy. Methods to reduce the pain caused by the flexible cystoscope passing through the urethral membrane appeared the key aspect.

In the above studies, patients were allowed to undertake deep breathing, observe closed-circuit television, and listen to music, in essence, to relax the external sphincter and reduce friction of the urethra membrane. The same effect is achieved by squeezing saline bags. In this study, we reasoned that further methods to relax the external urethral sphincter may further relieve the pain and discomfort experienced by patients. Anatomically, the urethral membrane is surrounded by an external urethral sphincter belonging to the striated muscle, which functions to control urination. In urodynamic studies, the external urethral sphincter is continually contracting during normal urine storage. At the beginning of urination, the external urethral sphincter relaxes, the urethral pressure drops, the bladder pressure rises, and urine is expelled from the urethra. Adults with normal nervous system functionality can initiate or suppress urination at will, even when the bladder contains only small amounts of urine. The cerebral cortex can also relieve inhibition of the urination center of the pons and urinate [19]. This provided a theoretical basis for this study. The maximum urethral pressure in urodynamics is equivalent to the urethral pressure at the external sphincter of the urethra. The maximum urethral pressure during adult male urine storage is 72.70 + 5.07 cmH2O, whilst the pressure of the external urethral sphincter decreases to 25-30 cm H2O during urination [12]. This provides a clinical basis for this study. The ability to actively control the cerebral cortex through active urination intention leads to relaxation of the external urinary sphincter and would allow the cystoscope to pass more smoothly. In our trial, we demonstrated that the VAS score among male patients who urinated during flexible cystoscopy was significantly lower. The mean VAS score had a decrease from 3 to 2. Whilst this study, we did not observe obvious relaxation of external urethral sphincter, the patient pain scores were significantly lower than those of the control group. In was likely that patients were attempting to prevent voluntary or reflex activation of the striated sphincter that occurs as a response to apprehension of catheterization and/or as a reflex response to afferent pudendal reflex arc stimulation, compared to if the reflex relaxation typically occurred without detrusor activation.

Thus, we have demonstrated that urination during flexible cystoscopy can significantly reduce cystoscopy induced pain in the urethra. Similarly, Stav K permits patients to perform urination during male catheterization, which contributes to relieving urethral pain [20].

In this study, the patient’s pain score decreased from 3 (IQR 2–3) to 2 (IQR 1–3), *P* = 0.001. Explain that our experiments have worked. However, from the clinical point of view, the extent of pain reduction is still small, and the pain score of cystoscopy itself is not high, so the clinical significance is small. We believe that this test can be used in rigid cystoscopy which can causes severe pain in the future to evaluate the clinical effect of this method.

This study has the following advantages: In addition to prospective, random controls, all operations were performed by the same highly skilled physician, thus maintaining consistency. Secondly, the characteristics of the two study groups were similar and both groups underwent comparable examination times. Thus, bias and other confounding factors were reduced to a minimum. The limitations of this study included the uncertainty that patients in the control group displayed no autonomous urination. In addition, apart from the subjective pain score, we had no objective evidence that the external urethral sphincter relaxed. Future studies should employ external urinary sphincter electromyography to ensure an objective evaluation of our study findings.

## Conclusions

In summary, we suggest that during male cystoscopy, patient urination represents a means of reducing pain levels during the examination.

## Data Availability

The datasets during and/or analyzed during the current study available from the corresponding author on reasonable request.

## Abbreviation

VAS: Visual analogue scale
